# Three-Dimensional Stratification of Bacterial Biofilm Populations in a Moving Bed Biofilm Reactor for Nitritation-Anammox

**DOI:** 10.3390/ijms15022191

**Published:** 2014-01-29

**Authors:** Robert Almstrand, Frank Persson, Holger Daims, Maria Ekenberg, Magnus Christensson, Britt-Marie Wilén, Fred Sörensson, Malte Hermansson

**Affiliations:** 1 Department of Chemistry & Molecular Biology, Microbiology, University of Gothenburg, Box 462, Göteborg SE-405 30, Sweden; E-Mail: fred.sorensson@cmb.gu.se; 2 Water Environment Technology, Department of Civil and Environmental Engineering, Chalmers University of Technology, Göteborg SE-412 96, Sweden; E-Mails: Frank.Persson@chalmers.se (F.P.); Britt-Marie.Wilen@chalmers.se (B.-M.W.); 3 Division of Microbial Ecology, Department of Microbiology and Ecosystem Science, University of Vienna, Vienna 1090, Austria; E-Mail: daims@microbial-ecology.net; 4 AnoxKaldnes AB, Klosterängsvägen 11A, Lund SE-226 47, Sweden; E-Mails: maria.ekenberg@anoxkaldnes.com (M.E.); magnus.christensson@anoxkaldnes.com (M.C.)

**Keywords:** moving bed biofilm reactor (MBBR), nitritation, anammox, AOB, wastewater, biofilm, stratification

## Abstract

Moving bed biofilm reactors (MBBRs) are increasingly used for nitrogen removal with nitritation-anaerobic ammonium oxidation (anammox) processes in wastewater treatment. Carriers provide protected surfaces where ammonia oxidizing bacteria (AOB) and anammox bacteria form complex biofilms. However, the knowledge about the organization of microbial communities in MBBR biofilms is sparse. We used new cryosectioning and imaging methods for fluorescence *in situ* hybridization (FISH) to study the structure of biofilms retrieved from carriers in a nitritation-anammox MBBR. The dimensions of the carrier compartments and the biofilm cryosections after FISH showed good correlation, indicating little disturbance of biofilm samples by the treatment. FISH showed that *Nitrosomonas europaea/eutropha*-related cells dominated the AOB and *Candidatus Brocadia fulgida*-related cells dominated the anammox guild. New carriers were initially colonized by AOB, followed by anammox bacteria proliferating in the deeper biofilm layers, probably in anaerobic microhabitats created by AOB activity. Mature biofilms showed a pronounced three-dimensional stratification where AOB dominated closer to the biofilm-water interface, whereas anammox were dominant deeper into the carrier space and towards the walls. Our results suggest that current mathematical models may be oversimplifying these three-dimensional systems and unless the multidimensionality of these systems is considered, models may result in suboptimal design of MBBR carriers.

## 1. Introduction

Nitrogen removal with anaerobic ammonium oxidizing (anammox) bacteria is an emerging technology for treatment of high strength wastewater. In the process, aerobic ammonia oxidizing bacteria (AOB) oxidize half of the ammonia to nitrite, which is used by anammox bacteria as electron acceptor for oxidizing the remaining ammonia to dinitrogen gas. In the more common full-scale configurations, the processes of nitritation and anammox occur in the same reactor [1]. The AOB and anammox bacteria can reside in suspended microbial assemblages as sludge and/or granules [2], or in biofilms attached to substrata [3]. One prerequisite for nitritation-anammox is the presence of both oxic and anoxic environments for AOB and anammox bacteria, respectively. This can be attained by alternating the environmental conditions in time, such as in a sequencing batch reactor [2]. In continuous reactors on the other hand, the microbial groups need to be separated in space, with anammox bacteria residing in anoxic zones of the microbial aggregates, provided by the oxygen demanding activities of AOB. In microbial granules, distinct patterns of stratification have been predicted by modelling and observed with microbiological methods, with AOB being located in the outer aerobic zone near the water interface and with anammox bacteria in the inner anoxic parts [4–8]. For nitritation-anammox biofilms on fixed substrata, the observed localization of microorganisms has been less straightforward. In several reactors, a seemingly scattered distribution of AOB and anammox bacteria has been observed [3,9–11], while in other reactors the distribution of microorganisms has been more orderly structured [12–14], indicating that the factors determining biofilm architecture are complex. Furthermore, there is a need for quantitative image analysis to reveal the actual distribution patterns of the biofilm microorganisms. For moving bed biofilm reactors (MBBRs), which are used today in full-scale nitritation-anammox [15,16], a recent investigation has indicated a more orderly biofilm structure [17], but detailed analysis of biofilm establishment and stratification is still lacking for these systems. One probable reason for this is the challenge to retreive and analyze undisrupted biofilms from the protected carrier compartments. Furthermore, biofilm modeling is a useful tool [18,19] to improve the efficiency of nitritation-anammox MBBRs. For these models a detailed knowledge about the distribution of microorganisms is fundamental.

In this study, we have utilized a cryosectioning approach for the retrieval and study of intact biofilm of different age from protected compartments of carriers residing in a nitritation-anammox MBBR. Fluorescence *in situ* hybridization (FISH) and confocal laser scanning microscopy (CLSM) was used to identify the AOB and anammox populations. By combining FISH-CLSM with a newly developed digital image analysis method [20], the three-dimensional stratification of AOB and anammox bacteria in intact biofilms was determined and quantified. Differences between young and mature biofilms in terms of established AOB and anammox populations and biofilm structure were investigated. Finally, image analysis of total carrier compartment *i.e.*, “wall-to-wall” biofilms, allowed an assessment of problems with biofilm retrieval and shrinkage from dehydration and cryo-embedding, which has often been suggested as a serious draw-back of these biofilm research methods.

## 2. Results and Discussion

### 2.1. Reactor Conditions

The nitritation-anammox MBBR was operated for three years. Our study covers 167 days at the end of the reactor runtime, from addition of new carriers to the final sampling. Reactor conditions during this period are summarized in Table 1. Since only trace amounts of organic carbon was added to the synthetic wastewater, and even though AOB in the biofilm could be a source for organic carbon *per se* [21,22], it is unlikely that denitrification would account for any significant nitrogen removal. The ratio of nitrate production to ammonia removal was 12%, which is close to the stoichiometric value for anammox bacteria of 11%, indicating that aerobic nitrite oxidation was not important [23].

### 2.2. Biofilm Retrieval and Sectioning

**Figure 1.**
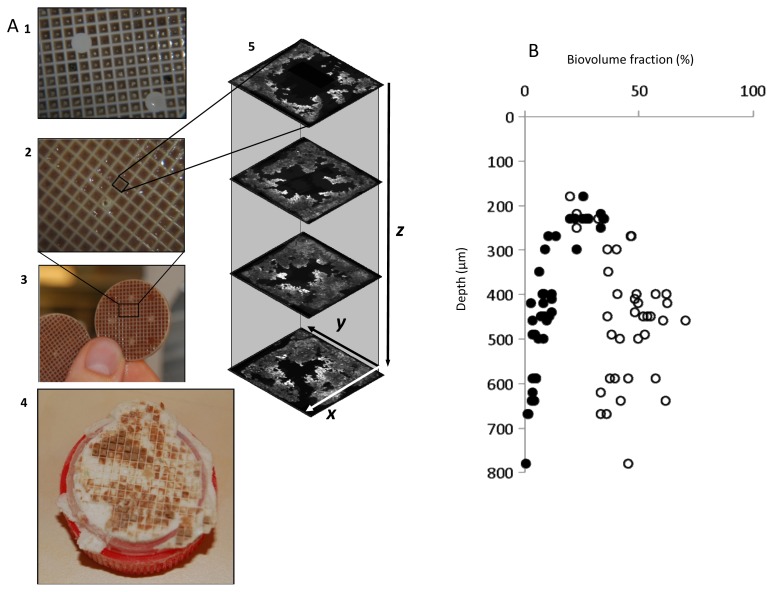
Overview of sample retrieval and analysis. Biofilm carriers containing either young (**A1**) or mature biofilm (**A2**,**3**) were cryo-embedded and frozen; After removal of the carrier, biofilm from carrier compartments was frozen solid and associated with the O.C.T. compound (**A4**); Cryosectioning of the biofilm was followed by FISH and CLSM analysis of biofilm stratification in the *x*, *y* and *z* dimensions (**A5**); and vertical (*z*) distribution from one of the compartments is exemplified in (**B**), anammox (open circles), AOB (closed circles).

Cryo-embedding, followed by freezing and removal of the carrier plastic material enabled the retrieval of biofilm from within individual carrier compartments (for carrier details see Table S1). As shown in Figure S1, the cryosectioned biofilm maintained the square shape in the *x*, *y* dimensions. The deepest analyzed cryosections (*i.e.*, in the *z* dimension, going into the carrier compartment) originated from at least 800 μm (Figure 1b). This corresponds to 80% and 53% of the maximum depth of the 2 and 3 mm carriers, respectively. Deeper layers can be analyzed, but long (in the *z* dimension) frozen biofilm structures can easily break. Previous studies [24,25] have observed that cryo-embedding and dehydration introduce severe distortion, such as shrinkage, to biofilm structure. Since the assembled micrograph squares represent a “wall-to-wall” *x*, *y* section of the biofilm (Figure 1a), and the fact that these were of the same dimension as the actual carrier compartment (Figure S1), we suggest that for these and similar types of biofilms, significant shrinkage is not introduced by the applied cryosectioning and hybridization protocols.

### 2.3. Bacterial Community Composition

A large FISH screening (Table S2) showed that the reactor community was made up of AOB in microcolonies and dense clusters of cells related to the *Nitrosomonas europaea/eutropha* lineage (cluster 7), hybridizing with the probe Nse1472. This was not unexpected considering the comparatively high ammonium concentrations in the reactor (average 314 mg N L^−1^). *N. europaea/eutropha-*related cells are generally encountered in systems with elevated ammonium concentrations [26] and may be regarded as AOB r-strategists with a comparatively high growth rate [27,28]. Simultaneous probe hybridization with Pla46 (targeting the order *Planctomycetales*), AMX820 (targeting the genera *Ca. Brocadia* and *Ca. Kuenenia*) and Bfu613 (targeting *Ca. Brocadia fulgida*) indicated that the anammox cells were related to *Ca. Brocadia fulgida. Ca. Brocadia*-like cells were also dominating the anammox population on biofilm carriers in a laboratory scale CANON reactor fed with concentrated anaerobic digestion reject water [29] and are possibly r-strategists among anammox bacteria [30]. Furthermore, filamentous cells targeted by probe CF319a (phylum *Bacteroidetes* among others), were rather evenly distributed, possibly providing structural support for the biofilm, as suggested earlier [31]. Very few NOB cells were present, as targeted by the probes for *Nitrobacter*, *Nitrospira* and *Nitrotoga* (Table S2), which was in accordance with the low aerobic nitrite oxidation (see above).

### 2.4. Biofilm Establishment on New Carriers

**Figure 2.**
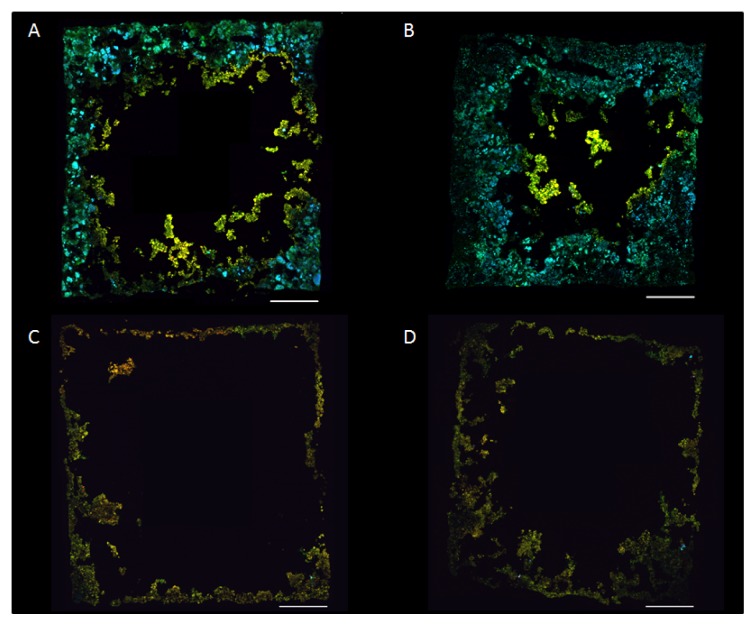
Assembled FISH “wall-to-wall” micrographs of biofilm from the biofilm carrier compartments. (**A**) Mature biofilm from 230 μm depth; (**B**) Mature biofilm from 500 μm depth; (**C**) Young biofilm from 250 μm depth; and (**D**) Young biofilm from 490 μm depth. In yellow, AOB cells hybridized with probe Nse1472. In cyan, anammox cells hybridized with probe Bfu613. In green, cells hybridized with the Eub338 (I–IV) probe mix only. Scale bar = 200 μm.

Anammox cells were almost completely absent from the relatively thin biofilms on the carriers incubated for five and a half months (Figure 2c,d). A few anammox microcolonies were found, usually in the compartment corners beneath biofilm dominated by approximately 80% of *N. europaea/eutropha*. Considering the DO concentration in the reactor (on average 3.6 mg L^−1^, Table 1), the results suggest a sequential colonization with initial formation of an AOB biofilm that with time created anaerobic microenvironments suitable for anammox bacteria in the deepest parts of the biofilm. It is likely that these environments first arose in the carrier corners, since the mature biofilms were thicker in the corners (336 ± 22 μm, average ± SD) than on the compartment sides (235 ± 19 μm). Thus, migration of intact AOB and anammox biofilm clusters alone cannot explain establishment of anammox on new carriers since AOB first needed to provide the anaerobic microenvironment. It has previously been shown that the desired stratification of AOB and anammox bacteria occurred only if anammox bacteria were established prior to AOB [13]. However, such orderly development was not necessary for stratification to develop here. The discovery of very few anammox bacteria after five and a half months suggests that the development took considerable time. Much faster establishment of anammox bacteria has been detected in anoxic reactors where AOB are not necessary to create anoxic microenvironments [32].

### 2.5. Biofilm Structure

As mentioned above, the biofilm was in general thicker in the corners than on the sides of the compartments. Biomass density, defined as the signal area of the Eub338 probe mix as a fraction of total compartment area (Figure S2), increased significantly with depth down to 400 μm (*R*^2^ = 0.4219, *n* = 17, α = 0.05). Thicker biofilms generally have a higher nitrogen removal, but modeling suggests improvements above a thickness of 750 μm to be small [33]. At more shallow depths (in the *z* dimension) erosion and detachment due to the shear forces, rather than electron donor or acceptor concentrations, may have controlled biofilm biomass. It was concluded that nitrifying biofilms on carriers fixed in a flow chamber generally showed enhanced erosion rather than growth when the flow rate increased, despite being supplied with more oxygen and substrate [19]. However, the relationship between carrier movement and the effect of flow velocity on biofilms in MBBRs is still uncertain [34].

**Figure 3.**
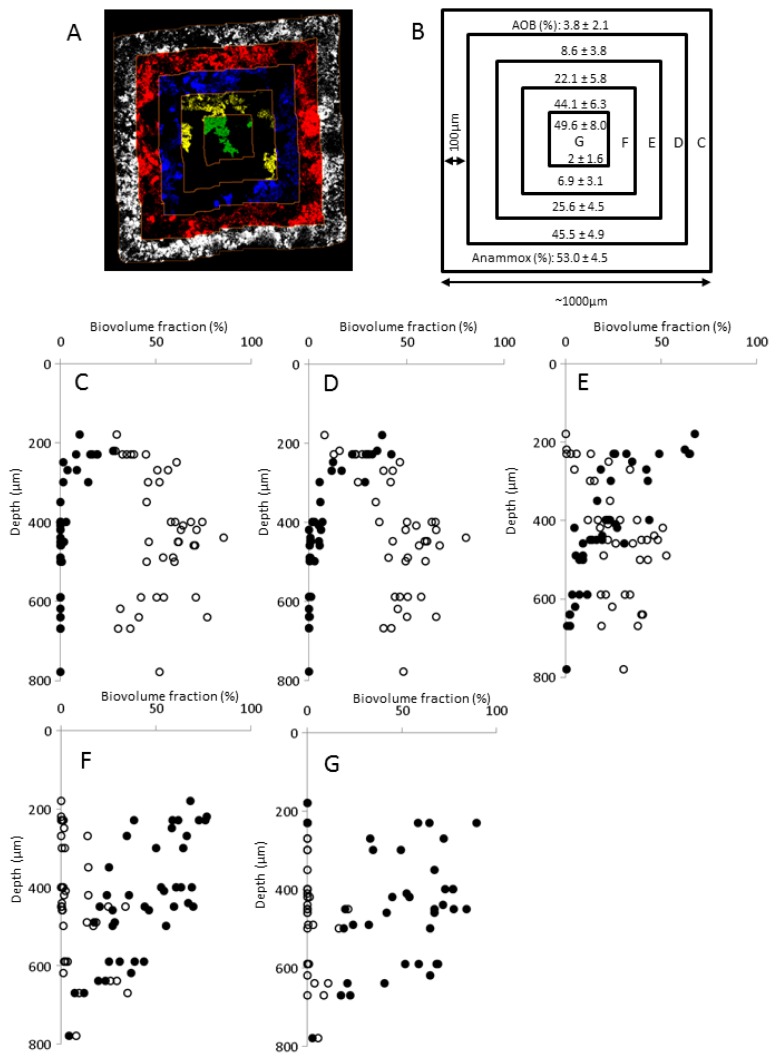
Assembled FISH “wall-to-wall” micrographs of biofilm from the biofilm carrier compartments in the *x*, *y* dimension. (**A**) Example of the multidirectional slicing procedure, where each colored area represents a 100 μm thick region of the biofilm from a particular distance from the compartment wall; (**B**) Schematic representation of the 100 μm thick slices, showing average biovolume fractions throughout all depths (*z*) of AOB and anammox bacteria in the carrier compartments. Average ± 95% confidence intervals, *n* = 41 (*n* = 37 for slice 400–500 μm); and (**C**–**G**) Biovolume fractions of anammox bacteria (open circles) and AOB (solid circles) in the concentric areas (colored “slices”) at different depths (*z*) in the carrier compartments. The designations (**C**–**G**) refer to the concentric areas at different distance from the compartment wall, as depicted in (**B**).

The overall vertical distribution in the mature biofilms showed a significant decrease of AOB with carrier compartment depth (*z* dimension) (*R*^2^ = 0.80, *n* = 41, α = 0.01; Figure 1b). Similar to AOB, anammox cells grew in characteristic clusters or microcolonies of varying size and shape (Figure 2a,b), but instead increased significantly in relative abundance with depth from 180 μm down to 400 μm (*R*^2^ = 0.66, *n* = 17, α = 0.01; Figure 1b). In the horizontal (*x*, *y*) dimension AOB dominated in the center parts and anammox in the peripheral part, close to the carrier walls (Figure 3). The least variation in relative population abundance was closer to the walls, such as for anammox in the 0–100 μm section (Figure 3c). Here erosion effects would be less severe compared with the center section where the variation of AOB was quite large between different compartments (Figure 3g). The combination of FISH, CLSM and new image analysis approaches enabled, for the first time, a detailed investigation of the three-dimensional distribution of AOB and anammox bacteria in MBBR biofilms. A uniquely detailed picture emerged, where the distinct stratification of AOB and anammox bacteria agrees well with the general pattern observed in granular biomass [4–6]. The decrease in relative abundance of AOB with carrier depth (the *z* dimension) is possibly caused by oxygen limitation in the deeper parts, whereas anammox bacteria increased down to 400 μm depth, below which the abundance leveled out or even decreased. This distribution pattern suggests different functional properties of the microbial communities at different depths of the biofilm in the carrier, where the outer regions contain mainly aerobic nitrite producers whereas the inner regions contain mainly anaerobic nitrite consumers. This kind of information is particularly valuable for understanding the function of MBBRs, since the continuous movement of the carriers in the reactors cause uncertainties about the flux of substrate and electron acceptors to the biofilm [34] and impede microelectrode *in situ* analysis of the gradients of substrate and electron acceptors near and in the biofilms [19].

## 3. Experimental Procedures

### 3.1. Reactor Conditions

A laboratory scale (7.5 liter) MBBR reactor with biofilm carriers (Minichip, AnoxKaldnes, Lund, Sweden) fed with synthetic wastewater (see Supplementary Information) was operated for three years. The reactor conditions and process performance are shown in Table 1.

### 3.2. Biofilm Sampling and Preparation

Characteristics of the biofilm carriers are listed in Table S1. New carriers were added to the reactor on December 17th 2008. Carriers were sampled for initial biofilm screening on May 4th, 2009. On June 2nd 2009, carriers containing young (5.5 months old) or mature (>18 months old) biofilms were sampled from the reactor. The carriers were immediately fixed in 4% ice-cold paraformaldehyde (PFA; pH 7.2) on ice for at least 8 h. After fixation, the carriers were submerged in phosphate buffered saline (PBS, pH 7.2) for at least 20 min and thereafter cut in halves and stored in 1:1 (PBS:EtOH) at −20 °C until further use.

For screening of biofilm community composition the biofilm was brushed off from fixed halves of biofilm carriers, fixed again and homogenized [35].

For intact biofilm analyses fixed halves of biofilm carriers were embedded in Tissue-Tek^®^ O.C.T.™ Compound (Sakura Finetek Europe B.V., Alphen aan den Rijn, The Netherlands), placed in a closed and parafilm-sealed Petri dish (Sarstedt, Helsingborg, Sweden) and incubated overnight at +4 °C. Centrifuge tubes (15 mL) were cut, placed upside down and filled to the brim with OCT compound. Incubated biofilm carriers were placed on top and covered with OCT and put in a liquid nitrogen fume chamber for approximately 60 min, until frozen solid. Tubes were fixed firmly in a vice and the carriers were removed, thereby exposing solid biofilm squares on top of the tubes (Figure 1a) which were sealed with a covering layer of OCT, frozen and stored in −70 °C. Sectioning of the biofilm from 15 compartments into 10 μm thick slices was performed in a cryotome operating at −20 °C. The produced sections were collected on SuperFrost^®^ Plus Gold microscope slides (Menzel GmbH & KG, Braunschweig, Germany) and stored in −20 °C.

### 3.3. Fluorescence *in Situ* Hybridization

Screening for AOB, NOB and anammox populations was performed on homogenized biofilms, sampled on 14 May 2009, using FISH [35].

On cryosectioned samples for intact biofilm analyses, OCT was removed by a 10 min submersion in 50% EtOH. Additional fixation, to ensure that all cells in the biofilm would be accessible to the fluorescent probes and FISH, was performed as described earlier [36,37]. The fluorescent probes and unlabeled competitors were obtained from Thermo Electron (Interactiva Division, Ulm, Germany) or MWG Biotech (Ebersberg, Germany). The probes were 5′ labeled with Cy3, Cy5, fluorescein or Alexa488. Hybridization was carried out at 46 °C for 2 (homogenized biofilm) or 4 h (cryosectioned biofilm), followed by washing at 48 °C for 10 min. Finally, the slides were rinsed in milli-Q water, air dried and mounted in Citifluor AF1 (Citifluor, London, UK). Each of the specific probes (Table S2) was hybridized together with the Eub338 probe mix for quantification of the relative probe signal.

### 3.4. Confocal Laser Scanning Microscopy

Confocal micrographs were acquired using a Bio-Rad Radiance 2000 MP microscope (Bio-Rad, Hemel Hempstead, UK) with a Nikon Plan Fluor 40×/1.40 oil objective, a Red diode laser (638 nm), a He/Ne laser (543 nm), and an Argon laser (488 nm). Images for analysis of biofilm structure were collected with the bundled software LaserSharp 2000 as 8-bit images of 1024 × 1024 pixels (resolution: 3.30 pixels/μm), and Kalman filtration (*n* = 3). For quantification of biofilm populations in homogenized samples, 8-bit images of 512 × 512 pixels (resolution: 1.65 pixels/μm), Kalman filtration (*n* = 2) were acquired. The biovolume fractions were measured in 21 microscope fields from each replicate by acquiring three images from different planes in the *z* dimension for each randomly selected x and y position, as previously described [38].

### 3.5. Digital Image Analysis

For biofilm structure analysis, the acquired images from each section were assembled manually using Photoshop CS4 extended (Adobe Systems, San Jose, CA, USA) to obtain complete coverage of the carrier compartment biofilm in each image (*i.e.*, “wall-to-wall” images). Here biofilm dimensions were measured to estimate shrinkage of the biofilm during cryosectioning, and biofilm thickness was measured (for details, see Supplementary Information). The biofilm images were then exported to the daime software version 2.0 [39] for measurements of the biovolume fractions of probe targeted bacteria in both homogenized and cryosectioned biofilm samples, and for measurements of the stratification in cryosectioned biofilms using the recently developed “Slicer” tool [20].

## 4. Conclusions

The applied cryosectioning and FISH protocols permitted retrieval and microscopy of intact biofilm from the MBBR carrier compartments with little biofilm distortion.

FISH data showed that *N. europaea/eutropha*-related organisms were the dominant AOB and that *Ca. Brocadia fulgida*-related organisms were the predominant anammox bacteria, suggesting a sequential colonization pattern of newly introduced carriers. Initial dominance of AOB was followed after about five and a half months by the establishment of anammox cells, probably as a result of formation of local anoxic micro-habitats by AOB.

By the use of novel digital image analysis tools and statistical analyses, substantial three-dimensional stratification of the mature biofilm was observed. In the *z*-dimension, the relative abundance of AOB decreased rapidly with depth, possibly due to oxygen limitation in the deeper parts of the carrier. In contrast, anammox bacteria increased in relative abundance down to a depth of 400 μm. A distinct horizontal (*x*, *y*), non-random distribution pattern was also observed, with AOB generally being most abundant in the center and anammox bacteria closer to the walls of the carrier compartments. We conclude that a true three-dimensional biofilm stratification can be expected in MBBR carrier systems, which should be taken into account when modeling and optimizing reactor performance and in the design of new MBBR carriers.

## Supplementary Information

### 1. Reactor Medium

The synthetic reactor media consisted of the following per 100 L: 240 g NaHCO_3_, 120 g NH_4_Cl, 0.3 g pepton, 0.56 g KH_2_PO_4_, 70 mL 2M NaOH, 40 mL micro-nutrient solution consisting of (in g/L): 4.8 MgSO_4_·7H_2_O; 1.6 MnCl_2_·2H_2_O; 5.8 CaCl_2_·2H_2_O; 0.48 CoCl_2_·6H_2_O; 0.24 NiCl_2_·6H_2_O; 0.26 ZnCl_2_; 0.10 CuSO_4_·5H_2_O; 1.44 FeCl_2_·4H_2_O; 0.0005 BH_3_O_3_; 0.0022 Na_2_MoO_4_·2H_2_O; 0.00114 Na_2_SeO_3_·5H_2_O; 0.0014 Na_3_WO_3_·2H_2_O.

### 2. Measurement of Biofilm Carrier Compartment Size

The biofilm carrier compartment dimensions were measured using brightfield microscopy and an eyepiece grid. The area was measured at the surface, halfway to the center and at the center, *i.e.*, the point farthest from the carrier surface. Note that due to the production method the carrier area decreases with depth down to the mid-point of the carrier. That is, the total carrier compartment, from one carrier surface to the other, has a slight “waist” at the center.

### 3. Digital Image Analysis of Biofilms

#### 3.1. Estimation of Biofilm Shrinkage

For estimation of biofilm shrinkage due to the cryosectioning and FISH protocols, the sides and diagonals of each assembled biofilm square were measured on the Eub338mix images, using the ruler tool in Photoshop (CS4 extended; Adobe Systems, San Jose, CA, USA). The dimensions were plotted and compared to the dimensions of the carrier compartments as determined by microscopy (see section 2 above).

#### 3.2. Estimation of Mature Biofilm Thickness

For estimation of the mature biofilm thickness, 8 measurements, one from each side and corner of each Eub338mix image were recorded in Photoshop CS4. Obvious biofilm channels were ignored in the measurements.

#### 3.3. Estimation of Biovolume Fraction of Populations

For estimation of the biovolume fraction of each population, an intensity threshold was set manually for each image, using the threshold function in Photoshop CS4. The resulting binary (black and white) images of probe-labeled biomass were exported as 16 megapixel TIFs to the digital image analysis software daime [39], where a noise reduction step was performed (“Noise reduction” function, number of non-zero neighbor pixels: 1). Due to the large size of the images, image resolution was reduced to 1 megapixel before measuring biovolume fractions. Following 2D segmentation of the binary images autofluorescent objects present in both the Nse1472 and Bfu613 images were removed. The biovolume fraction was calculated for each image after an additional step of artefact elimination [with the daime artifact rejection tool using a 50% congruency threshold and final congruency requirement between the specific probes and the biofilm reference (EUB338mix) of at least 90%]. Images of homogenized biofilm were analyzed through initial intensity thresholding (cutoff: 30), followed by RATS-L segmentation, followed by artifact elimination and calculation of the biovolume fraction of each specific probe-target population as part of the Eub388 probe mix with a >90% requirement for total congruency. The horizontal “wall-to-wall” population distribution was analyzed using the multidirectional Slicer tool in daime 2.0 [20], creating 100 μm thick virtual slices and applying a baseline smoothing of 20%. Relative population abundances were estimated for each virtual slice as described above.

**Table S1:** Characteristics of biofilm carriers. *

Carrier specifics	Type 1 1	Type 2 1
Depth of carrier compartments ( *i.e.*, *z* dimension in figures)	2 mm	3 mm
Diameter of the carrier	30 mm	30 mm
Protected surface area per carrier	2.73 × 10 ^−3^ m ^2^	4.10 × 10 ^−3^ m ^2^
Number of square compartments	325	325

* Minichip, AnoxKaldnes, Lund, Sweden.

1 Two different carrier variants were used.

**Table S2:** Oligonucleotide probes used for fluorescence *in situ* hybridization (FISH) in this study.

Probe name 1	Target	Sequence (5′–3′)	FA 2 [%]
Eub338 3	*Most Bacteria*	GCT GCC TCC CGT AGG AGT	0–50
Amx820	Anaerobic ammonium-oxidizing bacteria, *Candidatus* “ *Brocadia anammoxidans*” and *Candidatus* “ *Kuenenia stuttgartiensis*”	AAA ACC CCT CTA CTT AGT GCC C	40
Apr820	*Candidatus Anammoxoglobus propionicus*	AAA CCC CTC TAC CGA GTG CCC	40
Ban162	*Candidatus Brocadia anammoxidans*	CGG TAG CCC CAA TTG CTT	40
BS820	*Candidatus Scalindua wagneri*, *Candidatus Scalindua sorokinii*	TAA TTC CCT CTA CTT AGT GCC C	20
Bfu613	*Candidatus Brocadia fulgida*	GGA TGC CGT TCT TCC GTT AAG CGG	30
Sca1309	Genus *Candidatus Scalindua*	TGG AGG CGA ATT TCA GCC TCC	5
Scabr1114	*Candidatus Scalindua brodae*	CCC GCT GGT AAC TAA AAA CAA G	20
Pla46	*Planctomycetales*	GAC TTG CAT GCC TAA TCC	30
Nso1225	Most beta-proteobacterial AOB	CGC CAT TGT ATT ACG TGT GA	35
Nse1472	*Nitrosomonas europea*, *N. halophila*, *N. eutropha*, *Kraftisried-Isolat Nm103*	ACC CCA GTC ATG ACC CCC	50
Ntspa662 4	Genus *Nitrospira*	GGA ATT CCG CGC TCC TCT	35
Ntspa1151	Sublineage II of the genus *Nitrospira*	TTC TCC TGG GCA GTC TCT CC	35–40
Ntspa1431	Sublineage I of the genus *Nitrospira*	TTG GCT TGG GCG ACT TCA	35
Ntg840	*Nitrotoga arctica*	CTA AGG AAG TCT CCT CCC	10–20
Nit3 4	Genus *Nitrobacter*	CCT GTG CTC CAT GCT CCG	40
NmII	*Nitrosomonas communis* lineage	TTA AGA CAC GTT CCG ATG TA	35
Alf968	*Alphaproteobacteria*, except of *Rickettsiales*	GGT AAG GTT CTG CGC GTT	20
CF319a	Most *Flavobacteria*, some *Bacteroidetes*, some *Sphingobacteria*	TGG TCC GTG TCT CAG TAC	35
Gam42a 4	*Gammaproteobacteria*	GCC TTC CCA CAT CGT TT	35

1 For probe specifications, see ProbeBase (http://www.microbial-ecology.net/probebase/) [40];

2 FA = Formamide;

3 Used in a mix together with Eub338 II, III and IV; and

4 Used together with an unlabeled oligonucleotide competitor as indicated in the reference.

Figure S1 Scatter plot of area *versus* depth (*z*) for the assembled confocal micrographs (open circles) and Microchip compartment area as determined by light microscopy (closed circles). N.B. due to the production method the carrier area decreases with depth down to the mid-point of the carrier. That is, the total carrier compartment, from one carrier surface to the other, has a slight “waist” at the center. The area of the confocal micrographs was measured in the biofilm reference (Eub338) channel. Error bars = 95% confidence interval.

Figure S2 Scatter plot of total biomass areal density fraction *vs.* depth into the carrier. Each data point corresponds to the percentage coverage of the Eub338 probe mix, as determined by FISH, of the community in an assembled “wall-to-wall” micrograph from the indicated depth in the mature biofilm.

## Figures and Tables

**Table 1.** Reactor conditions and effluent concentrations during the experimental period. Average values and standard deviation (SD). The influent (synthetic wastewater) contained 314 mg N L^−1^ as ammonium. HRT = hydraulic retention time; DO = dissolved oxygen.

	Flow (L h^−1^)	HRT (h)	Temp (°C)	pH	DO (mg L^−1^)	NH_4_^+^ (mg N L^−1^)	NO_2_^−^ (mg N L^−1^)	NO_3_^−^ (mg N L^−1^)	N removal
Average	1.2	6.3	28.3	7.8	3.6	71	14	25	66%
S.D.	0.2	0.7	2.9	0.3	0.5	40	13	12	14%
