# Supplementary figures and images for: Three-Dimensional Stratification of Bacterial Biofilm Populations in a Moving Bed Biofilm Reactor for Nitritation-Anammox
Source: Int J Mol Sci. 2014 Jan 29;15(2):2191–206. doi: 10.3390/ijms15022191 (PMC3958845; doi:10.3390/ijms15022191)

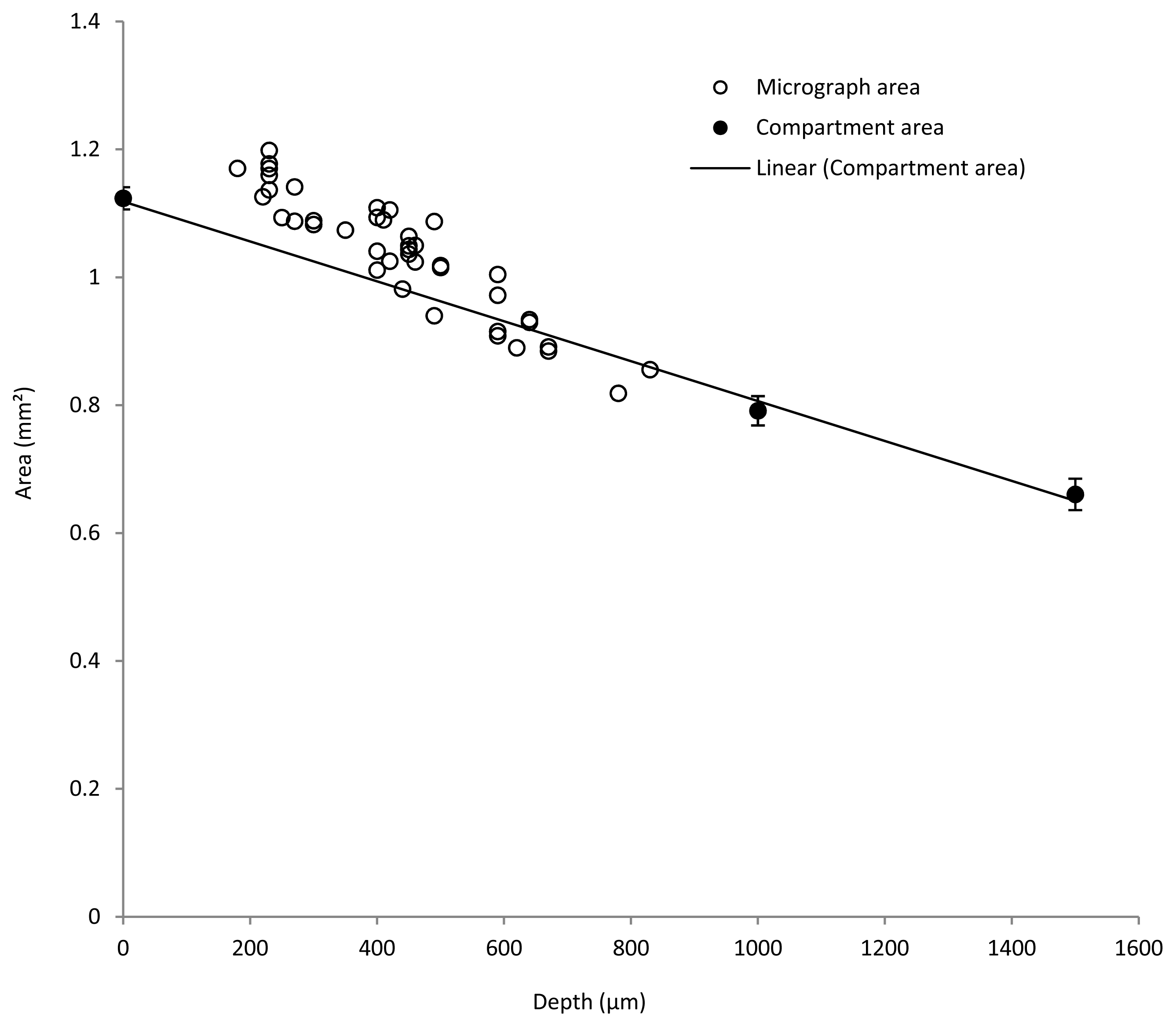

Supplement: Figure S1 — Scatter plot of area versus depth (z) for the assembled confocal micrographs (open circles) and Microchip compartment area as determined by light microscopy (closed circles). N.B. due to the production method the carrier area decreases with depth down to the mid-point of the carrier. That is, the total carrier compartment, from one carrier surface to the other, has a slight “waist” at the center. The area of the confocal micrographs was measured in the biofilm reference (Eub338) channel. Error bars = 95% confidence interval. [file ijms-15-02191s1.tif]

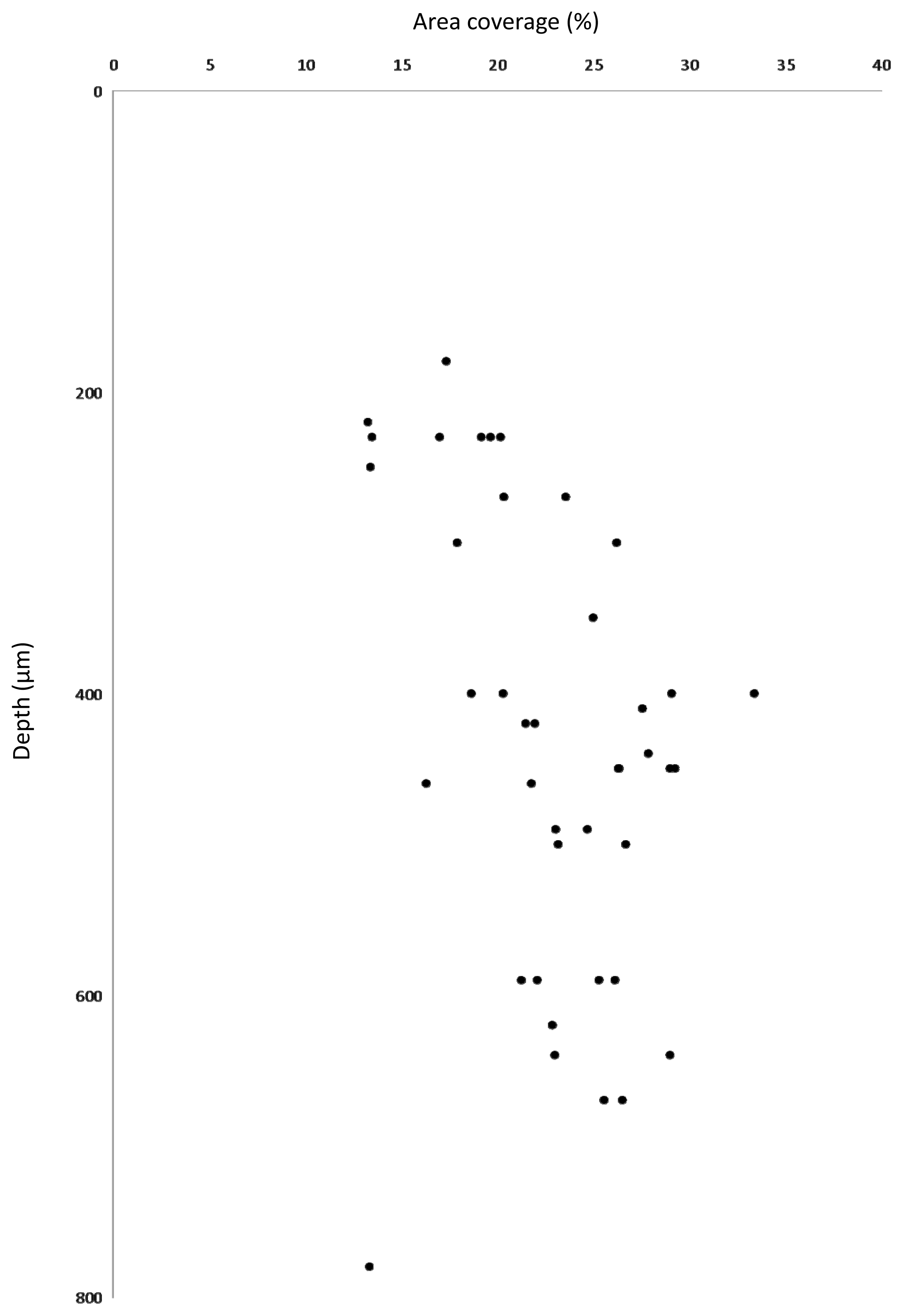

Supplement: Figure S2 — Scatter plot of total biomass areal density fraction vs. depth into the carrier. Each data point corresponds to the percentage coverage of the Eub338 probe mix, as determined by FISH, of the community in an assembled “wall-to-wall” micrograph from the indicated depth in the mature biofilm. [file ijms-15-02191s2.tif]
